# Lung, not only heart

**DOI:** 10.1186/2049-6958-9-21

**Published:** 2014-04-02

**Authors:** Annalisa Cogo

**Affiliations:** 1 Biomedical Sport Studies Center, University of Ferrara, Ferrara, Italy

## 

It is well known that physical exercise requires the coordinated function of the heart, lungs and peripheral and pulmonary circulation to support the increased demands of contracting muscles. This evidence is well represented by the so-called Wasserman’s gears representing the functional interdependence of the physiological components of the system and the mechanisms for coupling internal (i.e. cellular) and external (i.e. pulmonary) respiration [1]. The working muscles need a huge increase in oxygen utilization, which is achieved by increasing the extraction of oxygen from blood perfusing muscles and by increasing cardiac output, pulmonary circulation and ventilation.

The lung has therefore a crucial role in the performance of exercise but quite often this fact is neglected, especially in athletes (with exception of those suffering from exercise-induced bronchospasm) and in healthy subjects. Sometimes it seems that the role of the lung in exercise is considered only in subjects with respiratory limitations. Respiration is generally assumed to be the least important exercise-limiting factor because ventilation seems to have enough reserve. Indeed, the pulmonary system can be a limiting factor during exercise also in healthy asymptomatic subjects and not only in individuals suffering from cardiovascular and respiratory diseases. In particular, expiratory flow limitation and diaphragm fatigue can be detrimental to exercise performance in some endurance athletes [2]. Despite this fact, ventilatory evaluation has never been looked as an important tool for performance evaluation. By inserting in Pub Med the words “Exercise, Athletes, Heart” 1,188 papers are displayed in the last ten years; only 141 when “Heart” is replaced with “Lung”.

Furthermore, with regard to the exercise in athletes, the attention is almost always focused on the lung as gas exchanger rather than on the ventilatory parameters. Actually, the lung is composed of two functionally distinct compartments, equally important: the conducting region and the gas-exchange region, but this is the zone regarded with more interest when it comes to lung and exercise.

What is known about exercise and respiratory function? Some information is available about the influence of physical activity on pulmonary function. In fact, it has been shown that physical activity is associated with higher ventilatory parameters (especially FEV_1_) and the physiological decline in pulmonary function is slower in healthy subjects involved in regular, high intensity physical activity [3,4]. But very little or almost nothing is known about the opposite question: can a higher lung function affect the exercise capacity and performance?

The newest investigation from Di Paco and co-workers [5] has the merit of bringing attention to the respiratory function in relation to exercise performance in a group of elite soccer players, showing that the analysis of respiratory function and ventilatory parameters may be useful in the evaluation of exercise capacity in these athletes.

First, we learn that athletes that reach a higher maximal velocity during an incremental exercise test have a significantly greater forced expiratory volume in the first second and a greater peak ventilation. The athletes with higher ventilation also have a high ventilatory and cardiovascular efficiency as shown by the lower values of V_d_/V_t_ and the higher ratio VO_2peak_/HR.

A second interesting element is the development of the equation representing the significant relationship between VO_2_ peak and the ventilatory parameters, not only VE peak and breathing reserve but also FEV_1_. This opens a new perspective on the possibility of predicting the performance based on simple spirometric indices. Lung function assessment could then be fully included into the routine evaluation of the maximal work capacity of elite soccer players.

The last interesting topic, which is only mentioned and not fully developed in the paper, is the analysis of breathing pattern. In fact, a given ventilation can be achieved by a wide range of combination of tidal volume and respiratory rate and a breathing pattern characterized by a higher tidal volume and a lower respiratory rate for the same ventilation is more efficient [6]. A further analysis of breathing pattern throughout the incremental exercise test and not only at peak could give more valuable information in particular to define the “optimal” breathing pattern.

At the end of these considerations, a question arises: is it possible to exercise the ventilatory capacity? The well-known information about the effect of training on ventilation is the reduction of V_E_ for the same work rate, after an exercise training program [1], but not much is known about other specific breathing training. The first point concerns the respiratory muscles. The respiratory muscles are morphologically and functionally skeletal muscle whose primary task is to move the chest wall rhythmically to pump air into and out of the lungs. As breathing is a form of muscular exercise, it could also be trained. In fact, it has been shown that respiratory muscle training (both inspiratory muscle strength and respiratory muscle endurance training) improves endurance exercise performance and time trials in healthy individuals with greater improvements in less fit individuals and in sports of longer durations [7]. A meta-analysis demonstrated also a small albeit significant increase in FEV_1_ in athletes after respiratory muscle training compared with control [8]. Therefore, this type of training could be a step in the right direction. With regard to soccer, only one paper has examined the effect of respiratory muscle training concluding that this training improves intermittent exercise performance in soccer players [9].

Also a regular yoga practice improves pulmonary function in healthy individuals. As shown by a recent literature review [10] a minimum of 10 weeks of yoga practice improves maximum inspiratory and expiratory pressure, maximum voluntary ventilation, forced vital capacity and FEV_1_ in apparently healthy individuals, in eight out of nine studies. No information is available for athletes.

The second point concerns the breathing pattern and in particular the possibility of training individuals or athletes to adopt a more efficient ventilatory pattern, usually characterized by a deeper and slower breathing for the same ventilation. Up to now there are no research devoted to this topic in athletes.

The study by Di Paco and co-workers might pave the way for further research to analyze the role of respiratory parameters in the assessment of maximum exercise capacity in other sports besides soccer and to investigate the possibility of applying different types of specific respiratory training to improve dynamic ventilatory parameters and breathing strategies. And this is more intriguing for sport, like soccer, characterized by many change in activities, continually alternating running, sprint and jumps and requiring different ventilatory demands.
